# Ejaculate Economics: Testing the Effects of Male Sexual History on the Trade-Off between Sperm and Immune Function in Australian Crickets

**DOI:** 10.1371/journal.pone.0030172

**Published:** 2012-01-11

**Authors:** Damian K. Dowling, Leigh W. Simmons

**Affiliations:** 1 School of Biological Sciences, Monash University, Clayton, Victoria, Australia; 2 Centre for Evolutionary Biology, School of Animal Biology (M092), The University of Western Australia, Crawley, Australia; Arizona State University, United States of America

## Abstract

Trade-offs between investment into male sexual traits and immune function provide the foundation for some of the most prominent models of sexual selection. Post-copulatory sexual selection on the male ejaculate is intense, and therefore trade-offs should occur between investment into the ejaculate and the immune system. Examples of such trade-offs exist, including that between sperm quality and immunity in the Australian cricket, *Teleogryllus oceanicus*. Here, we explore the dynamics of this trade-off, examining the effects that increased levels of sexual interaction have on the viability of a male's sperm across time, and the concomitant effects on immune function. Males were assigned to a treatment, whereby they cohabited with females that were sexually immature, sexually mature but incapable of copulation, or sexually mature and capable of copulation. Sperm viability of each male was then assessed at two time points: six and 13 days into the treatment, and immune function at day 13. Sperm viability decreased across the time points, but only for males exposed to treatment classes involving sexually mature females. This decrease was similar in magnitude across both sexually mature classes, indicating that costs to the expression of high sperm viability are incurred largely through levels of pre-copulatory investment. Males exposed to immature females produced sperm of low viability at both time points. Although we confirmed a weak negative association between sperm viability and lytic activity (a measure of immune response to bacterial infection) at day 13, this relationship was not altered across the mating treatment. Our results highlight that sperm viability is a labile trait, costly to produce, and subject to strategic allocation in these crickets.

## Introduction

Historically, biologists considered that the discrepancy in gamete size between the sexes was a key factor that stood at the root of parental investment asymmetries, and conflicts, between the sexes. This idea was based on the assumption that since male gametes - the sperm - are tiny and their production seemingly endless, that they are therefore cheap to produce, at least much cheaper than their much larger female counterpart – the ova [1], [2].

The costs of reproduction [3], and sexual interaction [4], [5], [6], are well established in females, and represent one of the most fundamental of life-history trade-offs (that between investment in current reproduction relative to future reproductive and survival prospects) [7], [8]. While such costs were traditionally overlooked in males, there is now much evidence to demonstrate that they are considerable [9], [10], [11], [12], [13], [14], [15], [16].

Given such costs, it follows that males should face functional trade-offs when it comes to investment in post-copulatory reproductive processes [17]. Evidence for such trade-offs is fast accumulating, for instance between intra-ejaculate traits (e.g. size versus number) [16], between ejaculate allocation and mating acquisition [17], and ejaculate allocation and immune function [18], [19], [20], [21], [22]. In accordance with the idea that ejaculates are costly to produce, many examples exist of males differentially adjusting the quality or contents of their ejaculate in response to the perceived risk or intensity of sperm competition they face [17].

Initially, studies focussed on the capacity of males to differentially invest in the number of sperm transmitted per ejaculate [17], in accord with the original game-theoretical models by Parker and colleagues [23], [24] on ejaculate expenditure under different risks and intensities of sperm competition. Recently, however, it has become clear that promiscuity promotes intense sexual selection not only on sperm volume (sperm numbers, size), but also on traits that augment the quality of sperm (e.g. sperm motility, sperm viability) in the context of competitiveness [25], and on the composition of seminal fluid proteins within the ejaculate [26]. For example, in the Australian cricket (*Teleogryllus oceanicus*) the viability of a male's sperm makes a significant contribution to a male's reproductive success under sperm competition [27], and males can adjust the expression of this trait according to their perceived reproductive prospects [28], [29]. Thomas and Simmons [29] reported that male crickets can adjust their sperm viability in response to the mating status of females, with males producing ejaculates containing sperm of higher viability when mating to virgins or once-mated females than when mating to multiply-mated females. Furthermore, Simmons et al. [28] showed that males adjust their sperm viability according to the risk and intensity of sperm competition in this species.

Clearly then, sperm can be costly to produce, and males are able to strategically allocate their investment into the ejaculate. Moreover, female promiscuity invokes intense sexual selection on the male ejaculate, thus positioning the ejaculate as an ideal candidate trait to evolve under a parasite mediated model of sexual selection [30], [31]. Such models hinge on the idea that the expression of sexually selected male traits is constrained by competing investment demands on immune function. That is, a fundamental allocation trade-off should exist between investment in the ejaculate (pivotal to the male's reproductive success and under strong sexual selection) and into the immune system (pivotal to a male's survival prospects and under strong natural selection) [32]. Several studies have now provided evidence for sperm – immune trade-offs in the insects [18], [19], [20], including in *T. oceanicus* [21]. In particular, evidence is mounting that the upregulation of lysozyme – an enzyme integral to the innate immune system and defence against bacterial infection - is costly, resulting in reductions in ejaculate investment [20], [21].

In 2005, Simmons and Roberts [21] reported a negative genetic, and phenotypic, correlation between lysozyme (lytic) activity and sperm viability in *T. oceanicus*, consistent with the idea that the two traits are entwined in a fundamental allocation trade-off over reproductive investment and survival prospects. Indeed, it is plausible that this sperm – immune trade-off drives the patterns of strategic ejaculate investment, previously reported in this species [28], [29], in response to changes in perceived reproductive potential. Here, we aimed to examine the dynamics of this trade-off, exploring whether the costs associated with mating affect the expression of sperm viability in males, and whether the relationship between sperm viability and immune activity changes as the levels of sexual interaction that males experience are increased.

## Materials and Methods

### Stock population

All focal males used were recent descendants of a sample (n>100) of wild type adults, collected approximately one year previously from a banana plantation in Carnarvon, Western Australia. The laboratory stock population is maintained at large population sizes, propagated by hundreds of adults per generation via mass matings, and housed in a constant temperature room at 27°C on a 12 h light: 12h dark photoperiod.

*T. oceanicus* females are highly polyandrous and possess a negligible refractory period post-mating [33]. Females will readily remate within an hour of the first copulation.

### Experimental design

The experiment was conducted in four sampling blocks, each of which was separated in time by 14 days. In each block, virgin males were collected from the stock population at their penultimate moult, and stored in groups of 10 in 5 L plastic containers with *ad libitum* access to dry cat food and water. On the day of their final moult into adulthood, males were transferred to smaller individual holding containers (7×7×5 cm) with food and water, and aged in isolation for five to 12 days. All containers were cleaned twice weekly.

Each male was then randomly assigned to a *cost of mating* treatment consisting of three different classes. The treatment was applied to gauge the effects of increasing levels of sexual interaction on male sperm and immune quality. In each class, a single female was added to each male holding container, with food and water provided *ad libitum*. Experimental males assigned to the first class received female nymphs that were at their penultimate moult and therefore sexually immature. Such females do not respond to male courtship behaviours, and males do not actively court such females (based on tens of hours of personal observation). Males assigned to this class therefore experienced the lowest level of sexual interaction and are presumed to have produced the fewest spermatophores throughout the experimental sampling period. From hereon, we refer to this first class as the *sexually deprived* class.

Experimental males assigned to the second class received sexually mature adult females (10 days since their adult moult), but whose sub-genital plates had been blocked by application of a thin layer of superglue. Although this class of females responded to male courtship behaviours by attempting copulation, copulation was obstructed in all cases, with males unable to transfer a spermatophore. Males of this class therefore engaged in courtship (courtship singing, alignment into mating position), but experienced low to moderate spermatophore production costs because they were unable to transfer their spermatophores to females. We expect that males in this class likely dumped at least some of their old spermatophores on occasion and produced fresh spermatophores, but we assume that spermatophore production was lower in this class than in the third class described below. From hereon we refer to this class as the *courtship* class.

Experimental males assigned to the third class received adult females (10 d old) that were reproductively mature and able to copulate. These females responded to male courtship and copulated readily, resulting in spermatophore transfer to the female. We presume that males assigned to this class experienced the highest levels of sexual interaction, encompassing frequent courtship behaviour and high rates of spermatophore production. From hereon, we refer to this class as the *courtship and mating* class, since females were highly receptive to frequent matings.

The females within each class were rotated between containers daily, such that each experimental male experienced a new and unfamiliar female every 24 hours. Females were recycled and used over 13 days (and in the case of the immature treatment class, prior to the females moulting into adulthood), before their replacement with new females of the appropriate age (i.e. at penultimate moult for the nymph class and 10 days old for the other classes).

### Sperm viability assays

We used the *Live-Dead Sperm Viability Kit* (Molecular Probes, Eugene, OR, USA) to assay the sperm quality of focal males at six (i.e. when males were 11 to 18 d old as adults) and 13 days (males were 18 to 25 d old) into the experimental sampling period. All sperm viability assays were conducted between 0800 and 1200 h. The kit contains two dyes - SYBR-14 and Propidium Iodide - that differentially stain live (green fluorescence) and dead (red fluorescence) spermatozoa respectively. It can therefore be readily used to gauge the proportion of sperm within an ejaculate that are alive and viable. This kit has been regularly used by behavioural ecologists under the assumption that the trait assayed – sperm viability - is closely tied to a male's reproductive quality [29], [34], [35]. This assumption has been verified in the study species in focus, *T. oceanicus* [27].

At day 6 and 13, a spermatophore was extracted from each male and its contents allowed to gently dissipate into 20 µl of Beadle saline (128.3 mM Nacl, 4.7 mM KCL, 23 mM CaCl_2_) on a glass slide. Five microlitres of the dissipated contents were then extracted and mixed with 5 µl of a 1∶50 dilution of 1 mM SYBR-14 on a new glass slide. This solution was incubated in the dark for 5 min, before 2 µl of Propidium Iodide was gently mixed in, and the solution incubated for a further 5 min. At this point, a cover slip was added and the slide observed using a standard fluorescein excitation optical filter at 200×magnification. The colour of the first 500 sperm observed on the slide was scored across multiple randomly-positioned fields on the slide. A proportion of the sperm stained both green and red. These doubly-stained sperm comprised 10.5% of the total sperm counted (n = 140 741).

Sperm viability per ejaculate was then calculated as the proportion of green sperm in the total sperm pool (i.e. no. green sperm / [no. of green + red + doubly stained sperm]).

### Immune function assays

In the afternoon of day 13, each experimental male was assayed for three immune parameters.

#### Protein assay

Hemolymph protein content has been shown to be a good predictor of disease resistance in crickets [36]. The amount of protein in the hemolymph was determined using the Biorad Protein Assay (Biorad, NSW, Australia). 2 µl of hemolymph was taken by inserting a sterilized needle into the hemocoel via the intersegmental membrane. The hemolymph was diluted immediately into 198 µl of phosphate-buffered saline at pH 7.4 (PBS) (Astral Scientific NSW, Australia) and stored on ice. 5 µl of the diluted hemolymph was added to duplicate wells of a 96 well microplate, alongside a standard curve of bovine serum albumin (BSA) ranging in concentration from 1mg/ml to 0.1mg/ml. 200 µl of a 1 in five dilution of filtered Bradford dye reagent was added to the wells containing the BSA and hemolymph samples and the absorbance read at 600nm in a M5 *Spectramax* microplate reader (Molecular Devices, Sunnyvale, CA).

#### Lytic activity

Lytic activity is the key factor in antibacterial immunity of crickets [37]. Lytic activity was measured in each male using another 4 µl sample of hemolymph from the same male, which was diluted in 20 µl PBS. 10 µl of diluted hemolymph was placed into duplicate wells of a 96 well microplate. 10 µl of 1mM sodium azide (Sigma Aldrich, NSW, Australia) was added to each well to inhibit phenol oxidase activity, followed by 80 µl of a 3mg/ml solution of *Micrococcus lysodekticus* (Sigma Aldrich, NSW, Australia) in PBS. The plate was incubated at 33°C for 2 hours and then the absorbance measured at 492nm in a temperature controlled M5 *Spectramax* microplate reader. Controls were also run on each assay plate containing PBS instead of diluted hemolymph. Lytic activity was expressed as the difference in absorbance between the control and sample expressed as a percentage of the control.

#### Bacterial challenge

Males were then challenged with a dose of *Serratia marcescens* bacteria (Southern Biological, Victoria, Australia). An overnight culture of *Serratia marcescens* was grown from a glycerol stock in nutrient broth (Bacto Laboratories, New South Wales, Australia) at 37°C and the OD_600_ was measured. This was then diluted with nutrient broth to a concentration of 1.35 million cells/10 µl, previously determined to be the LD_50_ dose for *Teleogryllus oceanicus*. 10 µl of diluted bacteria was injected into the abdomen of each cricket using a Hamilton syringe. The crickets were then kept in a constant temperature room and checked daily to establish their longevity following the bacterial challenge.

### Statistical analysis

The experiment investigates a cost of mating treatment on sperm viability in males, and its covarying effects on immune function. We analysed the data using a Type 3 Repeated Measures General Linear Model in SYSTAT v13. Sperm viability (proportion of the first 500 sperm counted that were alive) was the response variable in the model, and was arcsine transformed to fulfil the assumption of normality of model residuals. Sampling Day was the repeated term (sperm viability at 6 and 13 days old), Treatment, Adult Age upon entering the experiment (5 to 12 days) and Block were treated as fixed effects, and lytic activity, protein levels, and survival following the bacterial challenge (days, log transformed) were treated as fixed covariates in the model. Non-significant (at α = 0.1) three-way interactions were dropped from the final model.

## Results

### Correlations between immune traits

There were no interactive effects between the immune parameters and the treatment (GLMs, results not shown). Thus, we present a correlation matrix to show the phenotypic correlations between immune variables, across the entire dataset. In general, males with high lytic activity had longer lives following a bacterial challenge (Table 1).

**Table 1:** Pearson correlation matrix of the immune parameters.

	Lytic activity	Protein	Lifespan
Lytic activity	1		
Protein	−0.129	1	
Lifespan	**0.272**	0.092	1

Pairwise comparisons that are significant at a = 0.05, following Bonferroni correction, are emboldened.

### Cost of mating and sperm viability

**Figure 1:**
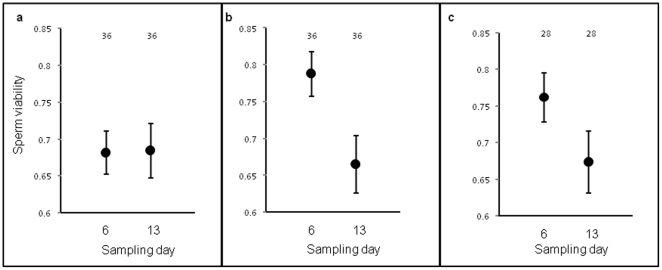
Age-dependent change in sperm viability across treatment classes. Each panel (a–c) denotes sperm viability (LS mean ±1 standard error) of males at day six and 13 of the experiment; a = males assigned to sexually deprived class, b = males assigned to courtship class, c = males assigned to courtship and mating class. Sample sizes per group are indicated above the bars.

Changes in sperm viability across the experiment (i.e. at Day 6 and 13) were contingent on the mating treatment to which males were assigned. Males assigned to treatment classes in which they had been exposed to adult females (*courtship* and *courtship and mating* classes) exhibited decreases in sperm viability with age, whereas *sexually deprived* males that had been exposed only to nymphal females exhibited low sperm viability throughout the experiment (Table 2, Figure 1). Thus, the cost of mating influences the expression of sperm viability in males.

**Table 2:** Repeated measures General Linear Model examining the cost of mating on sperm viability and covarying immune parameters.

Between subjects			
**Source**	*DF*	*F*	*P*
Treatment	2	1.176	0.314
**Block**	**3**	**5.650**	**0.001**
Days as adult	5	1.531	0.189
Lifespan after challenge	1	0.110	0.741
Lytic activity	1	0.045	0.832
Protein	1	1.307	0.256
Error	86		

Within subjects			
**Source**	*DF*	*F*	*P*
Sampling Day	1	0.561	0.456
**Sampling Day × Treatment**	**2**	**3.254**	**0.043**
Sampling Day × Block	3	0.784	0.506
Sampling Day × Days as adult	5	1.627	0.161
Sampling Day × Lifespan	1	0.08	0.778
Sampling Day × Protein	1	0.429	0.514
**Sampling Day × Lytic activity**	**1**	**5.206**	**0.025**
Error	86		

General Linear Repeated Measures Model. Final model, where response variable is Sperm Viability (arcsine transformed), Sampling Day of sperm viability (day 6 and 13) is the repeated term, and all other variables are fixed effects or variates.

There was also an interaction effect between Sampling Day and lytic activity on sperm viability (Tables 2). In particular, there was no association between lytic activity, measured at Day 13, and sperm viability measured earlier at Day 6 (r^2^ = 0.00, β = −0.017, n = 118, p = 0.872), but a weak negative but significant association when viability was measured at Day 13 (r^2^ = 0.03, β = −0.25, n = 118, p = 0.034). There was no interaction between Sampling Day, lytic activity and the cost of mating treatment on sperm viability (F_2,90_ = 0.154, p = 0.857), or between any of the immune parameters and treatment on sperm viability (results not shown).

There was a general effect of Block on sperm viability (Table 2, LS means ± SE; Block 1–4: 0.84±0.07, 0.73±0.04, 0.63±0.04, 0.63±0.04).

## Discussion

Our study shows that the quality of sperm in male crickets changes throughout life, exhibiting a general decrease in viability over two sampling periods that were separated by seven days. However, this decrease was only apparent in males that engaged in frequent courtship behaviour with adult females. Males that received no exposure to adult females throughout their adult lives, instead being housed with nymphal females, exhibited lower sperm viability at both time points (day 6 and 13). Furthermore, the decrease in sperm viability occurred both for males exposed to females that were receptive to insemination, and females that were incapable of receiving a spermatophore, due to the application of a fine film of glue to their subgenital plates.

The fact that sperm viability generally declined with time suggests a cost to the production of high quality sperm in *T. oceanicus*. Furthermore, our results suggest that these costs are mediated largely by resources invested into pre-copulatory reproductive processes, given that the decline in sperm quality was similar in magnitude for males assigned to the *courtship* and *courtship and mating* classes. That is, the common distinguishing factor across both of these mating classes was that the males in each were constantly exposed to adult females, which provoked strong and incessant pre-copulatory behavioural responses (male courtship song and alignment on females in the mating position) by males to achieve copulations. In contrast, only males of the *courtship and mating* class were able to freely transfer their spermatophores to females, and this invokes a much higher rate of spermatophore production than in the other mating classes (about one spermatophore per hour in contrast to about one per day in the other classes) [38], [39], [40]. Yet, there was no evidence that this added post-copulatory expenditure by males in the *courtship and mating* class had an increased effect on the expression of sperm viability with increasing age.

Thus, although we have presented evidence that the spermatophores involved in the post-copulatory mechanisms of mating and fertilization are costly to produce (since sperm viability declined across our time points), they appear to be no more costly than the pre-copulatory processes (courtship behaviours) required to achieve copulations. There are two further points relevant to this interpretation. First, males in the *sexually deprived* group will have produced on average the same number of spermatophores as males in the *courtship* group, and therefore differences between these two groups are therefore not likely to be attributable to differences in post-copulatory investment [38], [39], [40]. Rates of spermatophore production and their autonomous removal do not vary between male crickets housed alone or housed with females made incapable of copulation [39], and male *Teleogryllus* without access to receptive females with which to transfer a spermatophore overwhelmingly produce just one spermatophore a day [38]. These spermatophores are manufactured before they attempt to attract a female – i.e. prior to calling via stridulation [38], [39]. If males are repeatedly unsuccessful in their attempts to copulate, they eventually autonomously remove old spermatophores [38] because these become dessicated and inviable. Thus, the costs of spermatophore production for males assigned to the *courtship* and *sexually deprived* classes, while not nullified completely, were much lower than in the *courtship and mating* class.

The second point we raise is that given males in the *courtship* class were unable to successfully complete copulation, they might have elevated their rate of pre-copulatory investment above the rate experienced by males in the *courtship and mating* class (where males will have entered into regular ∼60–70 minute periods of non-sexual activity following successful copulation, while producing new spermatophores [36]). This means that the average rate of male pre-copulatory investment is likely to have differed across these two treatment classes over the 13 days of the experimental treatment, making it difficult to make direct inferences about the costs of pre- versus post-copulatory reproductive investment on sperm quality. Nevertheless, in combination, our results provide strong support for the idea that investment into each of the pre-copulatory and post-copulatory phases of reproduction carries sizeable costs to males, reflected by a decrease in sperm quality across time. Thus, males are likely to trade-off pre- and post-copulatory investment against each other, consistent with recent findings across taxa [41], [42], [43], [44]. Indeed, in *T. oceanicus* there is evidence to suggest that males that invest in high quality courtship song have reduced sperm viability [45], consistent with the existence of such a trade-off.

Previous studies have provided strong evidence for the idea that males can invest strategically in their ejaculate, in terms of productivity [46] but also quality [25]. Recently, studies have shown that, in *T. oceanicus*, males can adjust the expression of sperm viability according to their perceived reproductive prospects [28], [29], [47]. In our study, males exposed to immature females throughout the experiment produced spermatophores of low sperm viability at both time points, with viability scores at day 6 reflecting those of day 13 males assigned to the other two mating classes. This result reinforces the idea that males will strategically adjust the quality of their sperm in response to environmental cues. Moreover, the size of these adjustments (around 10% in our study and in [28], and 4% in [29]), highlight that the expression of sperm viability in *T. oceanicus* is highly labile, responsive to social cues, and condition dependent.

Condition dependence of sperm viability indicates that its expression might come at a cost to the expression of other key life-history traits, since life-history evolution is grounded on the existence of such trade-offs [8]. Here, we screened for negative associations between the expression of sperm viability and three immune parameters, motivated by the knowledge that sperm-immune trade-offs have been observed previously in the insects [18], [19], [20], including in *T. oceanicus* [21], and that the trade-off between immunity and reproduction is central to some of the most prominent models of sexual selection [30], [31]. In particular, we aimed to determine whether the negative association previously reported between lytic activity and sperm viability in *T. oceanicus*, by Simmons and Roberts [21], was affected by the costs of mating to males.

We confirmed a biologically weak (β = −0.25), but statistically significant, association between sperm viability and lytic activity in our study, when each variable was measured after 13 days of the experimental treatment. This association, although weak, is likely to be biologically meaningful, given that the expression of lytic activity was positively correlated with a male's ability to survive a bacterial challenge. The correlation between lytic activity and ability to survive the bacterial exposure in our study is striking, given that lytic activity was assessed using a gram positive bacterium (*M. lysodectus*), while our bacterial challenge involved resistance to a gram negative bacterium (*S. marcescens*). Gram negative and positive bacteria are fundamentally different, typically because the latter lack an outer layer of complex lipopolysaccharides to their cell wall. This would suggest, then, that males in better condition are able to better resist two very different forms of pathogenic challenge. It seems plausible that trade-offs will be amplified as levels of physiological stress increase (i.e. as the currency [e.g. energy [48], hormones [49], capacity to resist free radicals [7]] underlying the trade-off becomes exhausted). Given this, it was therefore surprising that the strength of the association between sperm and immune quality did not change across the mating treatments, indicating that the trade-off is not sensitive to the physiological costs of mating. In sum, our data suggest that males of intermediate age (i.e. 10 to 23 days old, as in our study) were not putting their survival prospects in serious jeopardy when investing in their ejaculate. That said, Simmons [50] recently found that males subjected to an immune challenge when juvenile (10 µl of 0.1% lipopolysaccharide from *Serratia marcescens*), do produced ejaculates exhibiting lower sperm viability than control males, but only when reared on a food-restricted diet. This suggests that the trade-off between sperm quality and immunity will hinge on the nutrient status of the males. In our study, males were fed *ad libitum*, hence continually satiated. It is plausible that the negative association we found between sperm viability and lytic activity would have been larger if assayed under the stressful conditions of dietary restriction. If so, then nutritional constraints faced by males might alter the dynamics of the sperm – immunity trade-off with increasing levels of sexual interaction, an avenue that will be worth pursuing in future studies.
